# Dynamic radiographic angle changes in planovalgus foot correction among children with cerebral palsy

**DOI:** 10.1007/s00402-026-06206-6

**Published:** 2026-02-19

**Authors:** Ana Laura Arenas-Díaz, Agustín Barajas-Monterrey, Silvestre Fuentes-Figueroa, Erika Alejandrina Barrón-Torres, Clemente Hernández-Gómez, Carlos A. Guzmán-Martín

**Affiliations:** 1 Medical Staff, Shriners Hospitals for Children - Mexico, Mexico City, Mexico; 2 Medical Fellowship in Pediatrics Orthopedics, Shriners Hospitals for Children - Mexico, Mexico City, Mexico; 3 Research Programs Department, Shriners Hospitals for Children - Mexico, Mexico City, Mexico; 4 Doctorado en Ciencias Biológicas y de la Salud, Universidad Autónoma Metropolitana, Ciudad de México, México

**Keywords:** Cerebral palsy, Planovalgus foot, Calcaneal lengthening, Mosca technique, Radiographic angles, Orthopedic surgery, Pediatric deformity, GMFCS

## Abstract

**Background:**

Planovalgus foot is the most frequent foot deformity in children with cerebral palsy (CP), often impairing gait and functional mobility. Radiographic angular measurements are essential tools for assessing deformity severity and monitoring postoperative outcomes, yet their dynamic behavior after correction remains poorly characterized.

**Objective:**

To analyze pre- and postoperative variations of radiographic angles in spastic planovalgus feet surgically treated using Mosca’s calcaneal lengthening technique within a retrospective cohort, and to provide an educational reference for orthopedic residents regarding angular behavior and measurement interpretation.

**Methods:**

A retrospective, longitudinal, descriptive study was performed on 132 (128 patients; 4 bilateral cases) feet of children with CP (GMFCS I–V) undergoing calcaneal lengthening, with or without adjunctive procedures (reefing or arthrodesis). Standardized weight-bearing anteroposterior and lateral radiographs were analyzed at five time points (preoperative, immediate postoperative, 6 months, 1 year, and 2 years). The evaluated angles included: AP talocalcaneal (Kite’s), AP talo–first metatarsal, talonavicular coverage, calcaneus–fifth metatarsal (C5M), Costa–Bartani, Meary’s, lateral talocalcaneal, and calcaneal inclination. Descriptive statistics were applied to assess temporal evolution.

**Results:**

All radiographic parameters showed immediate postoperative improvement, confirming effective deformity correction. The AP talocalcaneal angle decreased from 31.6° preoperatively to 24.0° postoperatively, while the calcaneal pitch increased from 7.1° to 19.1°. Mild regression was observed in several angles particularly C5M and Meary’s at two years, likely reflecting growth-related and neuromuscular factors.

**Conclusion:**

Calcaneal lengthening provides measurable and durable radiographic correction in CP-related planovalgus foot, although partial regression of some parameters over time highlights the dynamic nature of the deformity. Radiographic angles remain reliable for postoperative monitoring and educational training, supporting their use as a standardized multi-angle assessment tool in the medium-term management of these patients.

**Supplementary Information:**

The online version contains supplementary material available at 10.1007/s00402-026-06206-6.

## Introduction

Cerebral palsy (CP) represents one of the leading causes of motor disability in children, primarily of orthopedic origin. It encompasses a group of permanent, non-progressive disorders that impair movement and posture, frequently resulting in functional limitations throughout development. The estimated incidence ranges from 1 to 3 cases per 1000 live births [1, 2, 29].

Planovalgus foot is the most prevalent lower limb deformity observed in children with CP, occurring in an estimated 25–30% of cases. It is especially common among patients with diplegic and quadriplegic patterns and is often associated with gait disturbances and reduced functional mobility [2–4].

These foot deformities are primarily attributed to an imbalance of muscular forces acting on an immature and developing skeletal system [5]. The condition is characterized by several anatomical alterations: plantar flexion of the talus, external rotation of the calcaneus relative to the talus, and lateral displacement of the navicular bone. Additionally, forefoot supination occurs in relation to the hindfoot. Collectively, these changes result in the collapse of the medial longitudinal arch, exposing the talar head and producing a prominent midfoot bulge that is often palpable during clinical examination [2, 4, 6].

While clinical examinations are essential for identifying structural abnormalities, weight-bearing foot radiographs provide objective and quantifiable data crucial for assessing the severity of the deformity. Key angular measurements obtained from both anteroposterior (AP) and lateral views are fundamental for diagnosing planovalgus foot, monitoring its progression over time, guiding treatment planning, and evaluating postoperative outcomes [1–7].

Despite the availability of multiple studies reporting radiographic angles used to evaluate planovalgus foot deformities in patients with cerebral palsy, few research has described how these angular parameters change following surgical intervention. Therefore, this study aimed to analyze the pre- and postoperative variations of radiographic angles in spastic planovalgus feet surgically treated using Mosca’s calcaneal lengthening technique within a retrospective cohort, in order to better understand their temporal behavior and clinical relevance. Additionally, this work was designed to serve as an educational and reference tool for orthopedic residents, providing a structured visualization of the key angular parameters used in the assessment and follow-up of planovalgus correction in children with cerebral palsy.

## Materials and methods

### Study design and population

This retrospective cohort study included pediatric patients with a diagnosis of spastic cerebral palsy who underwent surgical correction of planovalgus foot deformity at Shriners Children’s Mexico between 2014 and 2020. All patients were classified within levels I to V of the Gross Motor Function Classification System (GMFCS) and had a minimum clinical and radiographic follow-up of two years. Eligible participants were 11 to 18 years of age and presented a symptomatic, flexible planovalgus deformity causing pain, gait disturbance, or orthotic intolerance. Only patients with complete serial radiographs suitable for angular analysis were included.

Patients were excluded if they had: (1) non-spastic or mixed types of cerebral palsy (hypotonic, athetoid, dystonic, or ataxic forms); (2) other neurological or neuromuscular disorders (e.g., traumatic brain injury, myelomeningocele, muscular dystrophy); (3) a history of previous foot surgery on the affected limb; (4) rigid or fixed deformities not reducible on clinical examination; (5) forefoot supination requiring medial cuneiform osteotomy; (6) procedures outside the standardized surgical protocol, or (7) incomplete radiographic documentation across follow-up time points. This study was reviewed and approved by the Research and Ethics Committee of Shriners Children’s Mexico in accordance with applicable national regulations (approval number: CEI-2023-05).

### Surgical technique

All patients underwent calcaneal lengthening using the Mosca technique, performed by the same surgical team under regional anesthesia, aseptic conditions, and with tourniquet control.

A lateral approach was made at the level of the calcaneocuboid joint. After careful dissection of the peroneal tendon sheath, the peroneus brevis was identified and tagged with an absorbable suture. A controlled transverse calcaneal osteotomy was performed approximately 1.5–2 cm proximal to the calcaneocuboid joint and oriented parallel to the joint surface. Correction was achieved either by distraction alone or by insertion of a pre-shaped tricortical iliac allograft to lengthen the lateral column and correct forefoot abduction and hindfoot valgus. The construction was stabilized with two 1.6 mm Kirschner wires crossing the calcaneocuboid joint to prevent subluxation. The decision to use a structural graft or distraction alone was left to the surgeon’s discretion, depending on intraoperative deformity correction and bone quality. Intraoperative fluoroscopy was used to evaluate correction and navicular coverage. When uncoverage was < 30% and the anteroposterior talar–first metatarsal angle (AP Talo–1st MT angle) was within normal limits, a talonavicular reefing was performed. In contrast, when uncoverage exceeded 30% or degenerative changes of the talonavicular joint were present, a talonavicular arthrodesis was carried out. Because of consistent triceps surae contracture, all patients underwent gastrocnemius lengthening. Patients with forefoot supination requiring medial cuneiform osteotomy were not included in this series. At the end of the procedure, the peroneus brevis tendon was repaired, and the incision was closed in layers.

Postoperative management consisted of six weeks of immobilization in a below-knee cast, followed by a structured rehabilitation program including physical therapy and gait retraining.

### Radiographic evaluation

Radiographic assessments were performed at five standardized time points: preoperative, immediate postoperative, 6 months, 1 year, and 2 years postoperatively. All radiographs were obtained in a weight-bearing position, except for the immediate postoperative films, which were non-weight-bearing due to immobilization. A standardized imaging protocol was applied to ensure consistent positioning and beam orientation across all evaluations.The following angular parameters were analyzed:

- Anteroposterior (AP) Talocalcaneal Angle (Kite’s Angle).
- Talar–First Metatarsal Angle (AP Talo–1st MT Angle).
- Talonavicular Coverage Angle.
- Calcaneus–Fifth Metatarsal Line Angle (C5M Angle).
- Moreau–Costa–Bartani Angle (Medial Longitudinal Arch Angle).
- Lateral Talar–First Metatarsal Angle (Meary’s Angle).
- Lateral Talocalcaneal Angle.
- Calcaneal Inclination Angle (Calcaneal Pitch Angle).

All angular measurements were performed using digital radiographs by two independent observers blinded to the time point.

### Statistical analysis

Descriptive statistics were used to summarize clinical and demographic data. Quantitative variables are presented as medians and interquartile ranges (IQR), while categorical variables are expressed as frequencies and percentages. Statistical analyses were performed using the Statistical Package for the Social Sciences (SPSS), version 26.

## Results

The most commonly analyzed radiographic angles are summarized in Table 1, which illustrates the standard anteroposterior and lateral projections used to quantify forefoot abduction, hindfoot valgus, and medial arch collapse. In addition to the normal reference ranges, the table specifies the main clinical purpose or cause for evaluation of each angle highlighting the specific deformity component, biomechanical alteration, or postoperative change that each measurement is intended to assess. This framework provides the rationale for angle selection and supports their use as objective parameters for diagnosis, surgical planning, and follow-up in pes planovalgus deformity.

**Table 1:** Radiographic angles in the AP and lateral view commonly used to evaluate planovalgus foot in CP

Angle	Radiographic AP view	Angle	Radiographic lateral view
AP talocalcaneal (Kite´s) angle. It is formed by the intersection of the talus and calcaneus axes [4]. Normal Value: 20 to 40° Quantifies hindfoot valgus and forefoot abduction; excessive values suggest planovalgus deformity [4]		Moreau–Costa–Bartani (Medial Longitudinal Arch) Angle. This angle is formed by two lines along the medial side of the foot: one line connects the lowest point of the calcaneus to the lowest point of the talonavicular joint, and the second line extends from that talonavicular point to the inferior aspect of the first metatarsal [8] Normal Value: Around 120° [9] Quantifies medial arch height; increased values indicate planus deformity	
AP talar–first metatarsal angle (AP talo–1st MT). The angle between the talus and the first metatarsal. This angle indicates the forefoot’s abduction relative to the hindfoot [10]. Normal Value: 0° and 5° Reflects forefoot abduction; normalizes after calcaneal lengthening [10]		Meary’s Angle (Lateral Talar–First Metatarsal Angle). The angle formed between the long axis of the talus and the long axis of the first metatarsal in a weight-bearing lateral view [11]. Normal Value: Approximately 0° [11] Represents sagittal alignment; used to assess arch collapse and overcorrection	
Talonavicular Coverage/Uncoverage Angle, measures how well the navicular bone covers the talar head in an AP radiograph [9] Normal Value: <20% uncovered [15] Evaluates talonavicular congruence; persistent uncoverage predicts recurrence		Lateral Talocalcaneal Angle. It is measured between the axes of the talus and calcaneus on a lateral radiograph. This angle reflects the subtalar alignment in the sagittal plane [5, 8, 12]. Normal Value: 30° to 40° [12] Assesses subtalar sagittal alignment; decreased values indicate arch collapse in planovalgus deformity [23,24,25,26].	
Calcaneus–5th Metatarsal Line Angle. The angle between a line drawn along the lateral border of the calcaneus and a line along the lateral border of the fifth metatarsal [13] Normal Value: Close to 0° [4] Indicates lateral column alignment and push-off efficiency; elevation signals over-lengthening		Calcaneal Inclination (Pitch) Angle. The angle formed between the inferior calcaneus and the horizontal plane, which is the foot–floor interface, as seen on a lateral weight-bearing radiograph. This angle reflects the tilt of the calcaneus and serves as an indicator of arch height [9, 14] Normal Value: 18 to 30 °[14] Reflects calcaneal inclination; lower angles = arch flattening [26, 27, 28].	

Table 1. Radiographic angles in the anteroposterior (AP) and lateral views commonly used to evaluate planovalgus foot deformity in children with cerebral palsy. Each parameter is illustrated on representative radiographs obtained under standardized weight-bearing conditions. The AP view includes the talocalcaneal (Kite’s) angle, talar–first metatarsal (AP Talo–1st MT) angle, talonavicular coverage angle, and calcaneus–fifth metatarsal line (C5M) angle, which together assess forefoot abduction, midfoot alignment, and hindfoot valgus. The lateral view demonstrates the Moreau–Costa–Bartani (medial longitudinal arch) angle, Meary’s (lateral talar–first metatarsal) angle, lateral talocalcaneal angle, and calcaneal inclination (pitch) angle, reflecting sagittal plane alignment and medial arch morphology. Normal reference ranges are provided for each measurement to facilitate interpretation of deformity correction and postoperative outcomes. A more detailed version of this table is provided in the Supplementary Material, as it serves as an excellent educational resource for orthopedic residents and fellows in training.

### Clinical and demographic characteristics

A total of 132 feet (128 patients; 4 bilateral cases) with spastic cerebral palsy (CP) who underwent surgical correction for planovalgus deformity were analyzed. The mean age at surgery was 14.1 years (range, 12.5–15.7 years), and 59.8% of patients were male. Regarding the topographic distribution of CP, diplegia was the most frequent presentation (62.9%), followed by hemiplegia (17.4%), quadriplegia (12.9%), triplegia (6.1%), and monoplegia (0.8%). Functional status according to the Gross Motor Function Classification System (GMFCS) showed that levels I–III represented 69.7% of the cohort, whereas levels IV–V accounted for 30.3%.

### Surgical characteristics and postoperative management

The mean joint–osteotomy distance was 11.0 mm (range, 7.05–15 mm), and the mean osteotomy length was 7.6 mm (range, 5.5–28 mm). In 56.8% of cases, osteotomy was performed parallel to the calcaneocuboid joint. Bone grafts were used in 25% of procedures, while K-wire fixation was employed in 87.9% of cases. The talonavicular joint was managed by reefing in 62.1% and arthrodesis in 37.9% of feet, based on intraoperative findings. Postoperative immobilization consisted of fiberglass casts in 58.3% and splints in 41.7%, maintained for six weeks. During follow-up, reintervention was required in 15.2% of cases. The predominant indications were recurrence of the planovalgus deformity characterized by loss of medial arch height and infracorrection as described by Chi-An Luo and colleagues [15]. A detailed breakdown of intraoperative and surgical variables including osteotomy length, joint-osteotomy distance, graft usage, fixation method, and talonavicular management is provided in Supplementary Table 3. In addition, the specific types of reinterventions performed are summarized in Supplementary Table 4.

**Table 2:** Clinical, demographic, and surgical characteristics of the study cohort

Variable	*n* = 132	Variable	*n* = 132
Clinical and demographic characteristics			
Age at surgery, years (range)	14.1 (12.5–15.7)	Gender: Female	53 (40.2%)
		Gender: Male	79 (59.8%)
CP Topography – Diplegia	83 (62.9%)	CP Topography – Hemiplegia	23 (17.4%)
CP Topography – Quadriplegia	17 (12.9%)	CP Topography – Triplegia	8 (6.1%)
CP Topography – Monoplegia	1 (0.8%)		
GMFCS Level I	19 (14.4%)	GMFCS Level II	24 (18.2%)
GMFCS Level III	49 (37.1%)	GMFCS Level IV	36 (27.3%)
GMFCS Level V	4 (3.0%)		
Surgical characteristics			
Joint–osteotomy distance, mm (range)	11.0 (7.05–15)	Osteotomy length, mm (range)	7.6 (5.5–28)
Osteotomy parallel to calcaneocuboid joint	75 (56.8%)	Bone graft used	33 (25.0%)
K-wire fixation	116 (87.9%)	Other fixation methods	16 (12.1%)
Reduction method – Talonavicular reefing	82 (62.1%)	Reduction method – Talonavicular arthrodesis	50 (37.9%)
Postoperative immobilization – Fiberglass cast	77 (58.3%)	Postoperative immobilization – Splint	55 (41.7%)
Reintervention required during follow-up	20 (15.2%)		

Continuous variables are presented as means (range), and categorical variables as percentages

Data correspond to 132 operated feet from (128 patients; 4 bilateral cases) with spastic cerebral palsy who underwent calcaneal lengthening for planovalgus correction

The table is organized into two sections clinical/demographic and surgical characteristics to facilitate interpretation and align with the results section narrative

The analysis of angular measurements over time reveals significant changes in median values following surgical intervention for spastic planovalgus. Most angles showed immediate postoperative improvements, reflecting effective surgical correction.

In the anteroposterior view, the AP talocalcaneal (Kite’s) angle decreased from a median of 31.6° (IQR 22.3–39.0) preoperatively to 24.0° (20.0–28.0) immediately postoperatively, showing a mild increase to 29.5° (20.3–33.9) at 2 years. The AP talar–first metatarsal angle decreased from 22.0° (13.6–29.1) to 5.0° (2.0–10.0) after surgery, stabilizing at 9.6° (4.0–15.9) at 2 years. Similarly, the Talonavicular Coverage/Uncoverage angle improved from 32.4° (21.3–40.0) to 3.6° (0.0–10.1) immediately postoperatively, maintaining correction with a median of 8.6° (0.0–19.5) at 2 years. The Calcaneus–5th Metatarsal Line (C5M) angle decreased from 14.0° (10.3–20.0) to 4.0° (0.0–7.4) and remained near 7.0° (1.6–11.4) at 2 years, indicating sustained correction of lateral column alignment.

**Fig. 1:**
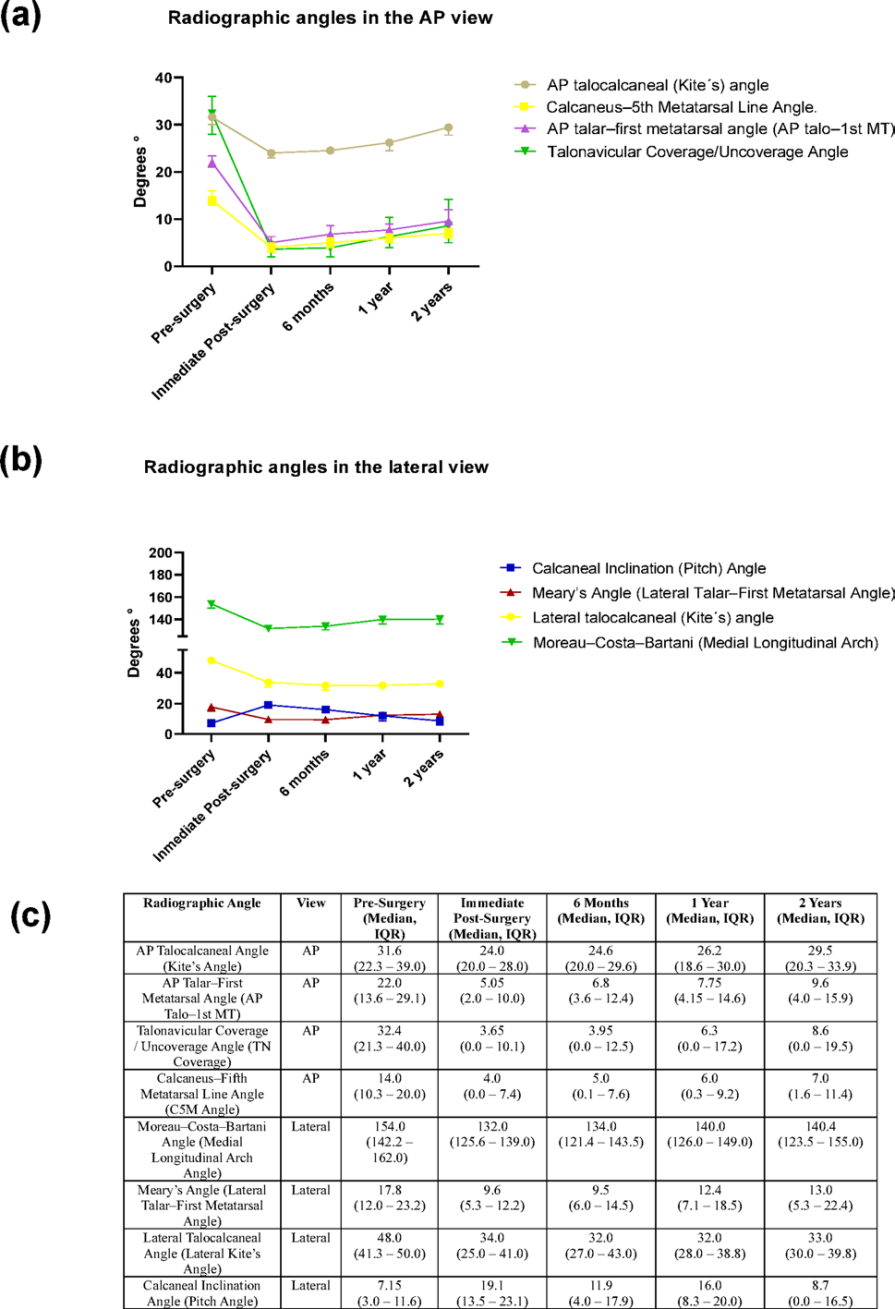
Temporal evolution of radiographic angular parameters following calcaneal lengthening for spastic planovalgus correction in children with cerebral palsy. **a** Radiographic angles measured in the anteroposterior (AP) view, including the AP talocalcaneal (Kite’s) angle, AP talar–first metatarsal (AP Talo–1st MT) angle, Talonavicular Coverage/Uncoverage angle, and Calcaneus–Fifth Metatarsal Line (C5M) angle. All AP parameters showed an immediate postoperative decrease, reflecting correction of forefoot abduction and hindfoot valgus, with mild regression and stabilization at two years. **b** Radiographic angles measured in the lateral view, comprising the Moreau–Costa–Bartani (Medial Longitudinal Arch) angle, Meary’s (Lateral Talar–First Metatarsal) angle, Lateral Talocalcaneal (Kite’s) angle, and Calcaneal Inclination (Pitch) angle. These parameters demonstrated marked improvement in sagittal-plane alignment and partial recovery of the medial arch, with minor regression over time but sustained correction relative to baseline. **c** Summary table presenting median (IQR) values for each radiographic angle across the five standardized time points (pre-surgery, immediate post-surgery, 6 months, 1 year, and 2 years). Collectively, the results confirm that calcaneal lengthening provides significant, durable improvements in both coronal and sagittal-plane alignment, despite slight postoperative remodeling during long-term follow-up

 In the lateral view, sagittal plane parameters also improved. The Moreau–Costa–Bartani (Medial Longitudinal Arch) angle decreased from 154.0° (142.2–162.0) to 132.0° (125.6–139.0) postoperatively and stabilized around 140.4° (123.5–155.0) at 2 years, reflecting partial restoration of the medial arch. The Meary’s angle improved from 17.8° (12.0–23.2) to 9.6° (5.3–12.2) immediately after surgery and remained within normal alignment ranges at 2 years (13.0°, IQR 5.3–22.4). The Calcaneal Inclination (Pitch) angle increased markedly from 7.1° (3.0–11.6) to 19.1° (13.5–23.1), with mild regression to 8.7° (0.0–16.5) at 2 years. Although some postoperative regression was observed across parameters between 6 months and 2 years, all angles remained improved relative to baseline values. These results confirm that calcaneal lengthening produces a durable yet dynamic correction in forefoot abduction, hindfoot valgus, and medial arch alignment. Figure 1; Table 2 depicts the temporal evolution of each angular measurement across the five standardized time points. Despite some postoperative regression, all angles remained improved compared to pre-surgical values, underscoring the overall effectiveness of the intervention and the importance of long-term monitoring to sustain corrections. These findings highlight the dynamic nature of surgical outcomes and the need for individualized follow-up care.

To facilitate interpretation, the temporal evolution of the radiographic angles is illustrated in Fig. 1, which depicts their behavior across all postoperative time points. In addition, two representative clinical cases from this cohort are presented in the Supplementary Material to exemplify the radiographic and clinical outcomes described in this study.

## Discussion

This study provides a valuable contribution to the orthopedic assessment and longitudinal monitoring of planovalgus foot deformity in pediatric patients with cerebral palsy (CP). By analyzing a retrospective cohort of 132 surgically treated feet with a two-year radiographic follow-up, we reinforce the diagnostic and prognostic utility of specific angular measurements and shed light on their dynamic evolution following surgical intervention.

Cerebral palsy remains the most common cause of pediatric motor disability, with planovalgus foot deformity representing a frequent challenge, particularly among ambulatory individuals [2]. These deformities can significantly impair gait, create pressure-related discomfort, and compromise functional mobility [16]. In our cohort, the prevalence of planovalgus was 62.9%, notably higher than that reported in prior studies, especially among patients with diplegia [2, 4, 6]. Most cases (69.7%) corresponded to GMFCS levels I - III, consistent with the literature [17].

While conservative treatment remains the first-line approach, including physical therapy and orthotic use, surgical correction is warranted when symptoms persist, or deformity progresses. All patients in our study underwent calcaneal lengthening using Mosca’s modification of the Evans procedure, supplemented with adjunctive techniques such as tendon balancing and midfoot stabilization. This comprehensive approach aims to correct hindfoot valgus, restore medial arch support, and reduce forefoot abduction [2, 3, 5, 17].

Notably, the average age at surgery was 14.1 years, which may have contributed to our lower recurrence rate (15.2%) compared to the 25% reported by others [17–19]. Younger age at surgery, especially under 10–11 years, is associated with higher risk due to the dynamic nature of planovalgus during growth and adolescence [15, 17]. Technique variation within the cohort, particularly the predominance of talonavicular reefing (62.1%) over arthrodesis (37.9%), provides an opportunity to examine their influence on long-term angular correction. Although both yielded satisfactory immediate results, long-term stability remains uncertain.

Factors such as osteotomy length (mean 11.0 mm), joint proximity, type of fixation (87.9% K-wires), and immobilization methods may also affect outcomes and warrant further investigation. Subgroup analyses are needed to clarify their role in angle preservation and deformity recurrence.

Radiographic assessments revealed significant angular correction immediately after surgery across all measured parameters. These included the AP talocalcaneal Angle (Kite´s angle), AP talar-first metatarsal Angle (AP talo-1st MT), Talonavicular Coverage/Uncoverage Angle, Calcaneus-5th Metatarsal Line Angle, Moreau-Costa-Bartani Angle (Medial Longitudinal Arch), Lateral Talar–First Metatarsal Angle (Meary’s angle), Lateral talocalcaneal Angle and Calcaneal Inclination Angle (Calcaneal Pitch angle). AP talocalcaneal (Kite’s) angle, AP talar–first metatarsal angle (AP talo–1st MT), talonavicular coverage angle, C5M angle, Costa–Bartani angle, Meary’s angle, lateral talocalcaneal angle, and calcaneal pitch. These findings corroborate prior literature that validates the role of these angles in both deformity classification and surgical planning. For example, the AP talocalcaneal Angle (Kite´s angle) decreased from a median of 31.6° preoperatively to 24° postoperatively, aligning with expected forefoot realignment. Likewise, the Calcaneus-5th Metatarsal Line Angle and Lateral talocalcaneal Angle demonstrated robust immediate corrections, confirming their sensitivity to surgical intervention.

In line with the findings of C. A. Turriago [30], who reported postoperative improvements in the AP talocalcaneal angle (Kite´s angle) and AP talar-first metatarsal Angle (AP talo-1st MT) following calcaneal lengthening without recurrence over a 40-month follow-up [20], our results also demonstrated initial correction. However, unlike Turriago’s series, we observed gradual angular changes over time, likely related to the progressive contracture of agonist and antagonist muscle groups during growth. This suggests that long-term follow-up is essential to detect subtle recurrences associated with neuromuscular imbalance.

On the other hand, J.S. Davitt et al. (2002) analyzed radiographic changes before and after calcaneal lengthening and reported significant improvements in both the Talonavicular Coverage Angle and the AP talar-first metatarsal Angle (AP talo-1st MT) [21].

Interestingly, at two years indicates the tendency of some angular parameters to deteriorate over time, possibly due to persistent neuromuscular imbalances or suboptimal foot stabilization. Our findings demonstrate that while radiographic improvements after flatfoot surgery in cerebral palsy patients are generally sustained, mild regression occurs over time, particularly in angles like the Calcaneus-5th Metatarsal Line Angle and the Lateral Talar–First Metatarsal Angle (Meary’s angle). This aligns with previous smaller studies and underscores the need for cautious interpretation of early postoperative success and long-term monitoring.

Unlike the Lateral Talar–First Metatarsal Angle (Meary’s angle), which has established thresholds guiding surgical decisions, the Calcaneus-5th Metatarsal Line Angle angle lacks a universal cutoff, though elevated values may indicate more severe deformity and inefficient push-off [7, 22, 23]. Our data show that the Calcaneus-5th Metatarsal Line Angle angle initially improves postoperatively but tends to return to abnormal values over time, highlighting the dynamic nature of neuromuscular deformities.

Similarly, the Moreau-Costa-Bartani Angle (Medial Longitudinal Arch), a marker of arch collapse severity, improved postoperatively but increased again over time, consistent with Ramírez-Barragán et al.’s findings [22]. Nicole Look’s 2021 work reinforces the importance of the Calcaneal Inclination Angle (Calcaneal Pitch angle) as a sensitive measure of deformity severity [3, 24]. In our cohort, this angle improved immediately after surgery but declined over time, reflecting the deformity’s complexity.

Lateral Talar–First Metatarsal Angle (Meary’s angle) remains a critical tool for assessing surgical outcomes and timing reintervention [3]. In our analysis, despite initial improvement after calcaneal osteotomy, the angle progressively worsened over two years, supporting Min et al.’s observations on deformity progression in children with higher GMFCS levels [3].

Our results also confirm that calcaneal lengthening effectively reduces the AP talar-first metatarsal Angle (AP talo-1st MT), a recognized surgical success indicator [22], and that Talonavicular Coverage improves significantly postoperatively and remains stable, correlating with improved foot loading patterns [9].

However, the dynamic behavior of these angles suggests that radiographic correction does not guarantee long-term biomechanical stability, which is crucial when considering reintervention or rehabilitation strategies. This is emphasized by our 15.2% reintervention rate and aligns with MacInnes et al.’s systematic review, which noted higher recurrence with calcaneal lengthening, especially in patients with advanced GMFCS levels [11].

Our findings align with and extend previous studies by offering a robust, multi-angle analysis across standardized follow-up intervals. Unlike prior work limited to single-angle assessments or short-term outcomes, this study captures the evolution of radiographic alignment in a sizable and diverse CP cohort.

The accompanying literature review affirms the relevance of each angle in the literature but also highlights a lack of standardization in measurement techniques and cutoff values. Our work addresses this gap by applying a consistent radiographic protocol across multiple angles and time points, serving as a potential reference for future studies and clinical evaluations.

### Limitations

This study has limitations that should be acknowledged. First, its retrospective design limits control over potential confounding factors and introduces an inherent risk of selection bias. Although all procedures adhered to the Mosca technique, important intraoperative decisions such as the use of bone grafts, fixation configuration, and the choice between talonavicular reefing or arthrodesis were left to the operating surgeon’s discretion. This inevitably generated heterogeneity in surgical management that may have influenced postoperative radiographic outcomes.

Second, no a priori sample size estimation was performed. This is because the present manuscript represents the descriptive and bibliographic phase of a broader, multi-stage research program. The objective at this stage was to document radiographic trajectories and develop a standardized framework for understanding angular behavior in pes planovalgus deformity. As a result, the study is intentionally exploratory. The lack of formal power calculation limits the inferential strength and generalizability of the findings; however, this foundational analysis is necessary to inform the methodological design of subsequent phases. Importantly, the detailed analysis of surgical revision cases including causes, risk factors, and predictive modeling for loss of correction will be incorporated in Phase II of the project and is intentionally beyond the scope of the present descriptive phase.

Third, radiographic evaluation relied exclusively on standard anteroposterior and lateral weight-bearing projections. While these are the conventional and reproducible views used in clinical practice, they do not capture the full three-dimensional complexity of hindfoot and midfoot deformities. Advanced modalities such as biplanar EOS imaging or weight-bearing CT may provide a more comprehensive spatial assessment and should be considered in future work.

Finally, although radiographic measurements were performed independently by two observers, formal inter- and intra-observer reliability analyses were not conducted. Incorporating reliability assessment in future phases will strengthen methodological rigor and improve reproducibility.

## Conclusion

This work represents the descriptive and developmental phase of a broader project aimed at constructing a standardized study tool for the evaluation of pes planovalgus deformity in children with cerebral palsy. Through a comprehensive bibliographic review of radiographic angles and their clinical significance, combined with a retrospective analysis describing their temporal behavior following calcaneal lengthening, this study reinforces the clinical and educational value of angle-based assessment for diagnosis, surgical planning, and postoperative monitoring. The consistent improvement observed across multiple angular parameters—particularly the AP talocalcaneal (Kite’s), AP talar–first metatarsal (AP Talo–1st MT), Calcaneus–Fifth Metatarsal Line (C5M), and Lateral Talar–First Metatarsal (Meary’s) angles—demonstrates the utility of these measures in quantifying surgical correction. Nevertheless, the gradual regression detected during mid-term follow-up underscores the deformity’s dynamic nature and the need for long-term surveillance. This manuscript establishes the foundational descriptive framework upon which subsequent work will build: a future inferential analysis incorporating larger prospective data and the development of a real-time predictive calculator designed to estimate key surgical and radiographic outcomes based on patient- and procedure-specific variables.

## Supplementary Information

Below is the link to the electronic supplementary material.

Supplementary Material 1

## Data Availability

Raw data will be provided with a reasonable request to the corresponding authors.

## References

[1] Bax M, Goldstein M, Rosenbaun P, Leviton A, Paneth N, Dan B et al (2005) Proposed definition and classification of cerebral palsy, April 2005. Dev Med Child Neurol 47(8):571 16108461 10.1017/s001216220500112x

[2] Sadowska M, Sarecka-Hujar B, Kopyta I (2020) Cerebral palsy: current opinions on definition, epidemiology, risk factors, classification and treatment options. Neuropsychiatr Dis Treat 16:1505–1518 32606703 10.2147/NDT.S235165 PMC7297454

[3] Min JJ, Kwon SS, Sung KH, Lee KM, Chung CY, Park MS (2020) Progression of planovalgus deformity in patients with cerebral palsy. BMC Musculoskelet Disord 21(1):141 32127007 10.1186/s12891-020-3149-0 PMC7055068

[4] Kadhim M, Holmes L, Church C, Henley J, Miller F (2012) Pes planovalgus deformity surgical correction in ambulatory children with cerebral palsy. J Child Orthop 6(3):217–227 23814622 10.1007/s11832-012-0413-3 PMC3400002

[5] Sung KH, Chung CY, Lee KM, Lee SY, Park MS (2013) Calcaneal lengthening for planovalgus foot deformity in patients with cerebral palsy pediatrics. Clin Orthop Relat Res 471(5):1682–1690 23179128 10.1007/s11999-012-2709-5 PMC3613565

[6] Ghanem I, Massaad A, Assi A, Rizkallah M, Bizdikian AJ, El Abiad R et al (2019) Understanding the foot’s functional anatomy in physiological and pathological conditions: the calcaneopedal unit concept. J Child Orthop 13(2):134–146 30996737 10.1302/1863-2548.13.180022 PMC6442506

[7] Aboelenein AM, Fahmy ML, Elbarbary HM, Mohamed AZ, Galal S (2020) Calcaneal lengthening for the pes planovalgus foot deformity in children with cerebral palsy. J Clin Orthop Trauma 11(2):245–250 32099288 10.1016/j.jcot.2018.07.021 PMC7026583

[8] Mosca VS (1995) Calcaneal lengthening for valgus deformity of the hindfoot: results in children who had severe, symptomatic flatfoot and skewfoot. J Bone Joint Surg 77(4):500–512 7713966 10.2106/00004623-199504000-00002

[9] O’Connell PA, D’Souza L, Dudeney S, Stephens M (1998) Foot deformities in children with cerebral palsy. J Pediatr Orthop 18(6):743–7 9821129

[10] Ramírez-Barragán A, Galán-Olleros M, Maroto R, Egea-Gámez RM, Martínez-Caballero I (2022) Long-term outcomes of talonavicular arthrodesis for the treatment of planovalgus foot in children with cerebral palsy. J Pediatr Orthop 42(4):E377–E383 35132016 10.1097/BPO.0000000000002081

[11] Mehanna J, Massaad A, Assi A, Rassi J, Atallah A, Ghanem I (2023) Risk factors for failure of calcaneal lengthening osteotomy in children and adolescents with planovalgus foot deformity: a retrospective study. Cureus. 10.7759/cureus.43157 37692710 10.7759/cureus.43157 PMC10484500

[12] Davitt JS, MacWilliams BA, Armstrong PF (2001) Plantar pressure and radiographic changes after distal calcaneal lengthening in children and adolescents. J Pediatr Orthop 21(1):70–75 11176357 10.1097/00004694-200101000-00015

[13] Sees JP, Miller F (2013) Overview of foot deformity management in children with cerebral palsy. J Child Orthop 7(5):373–377 24432097 10.1007/s11832-013-0509-4 PMC3838514

[14] Andreacchio A, Orellana CA, Miller F, Bowen TR (2000) Lateral column lengthening as treatment for planovalgus foot deformity in ambulatory children with spastic cerebral palsy. J Pediatr Orthop 20(4):501–5 10912608

[15] Kadhim M, Holmes L, Miller F (2012) Correlation of radiographic and pedobarograph measurements in planovalgus foot deformity. Gait Posture 36(2):177–181 22525421 10.1016/j.gaitpost.2012.02.011

[16] Look N, Autruong P, Pan Z, Chang FM, Carollo JJ (2021) Radiographic and plantar pressure assessment of pes planovalgus severity in children with cerebral palsy. Clin Biomech 85:105364 10.1016/j.clinbiomech.2021.105364 33940478

[17] Luo CA, Kao HK, Lee WC, Yang WE, Chang CH (2017) Limits of calcaneal lengthening for treating planovalgus foot deformity in children with cerebral palsy. Foot Ankle Int 38(8):863–869 28474963 10.1177/1071100717702596

[18] Polichetti C, Borruto MI, Lauriero F, Caravelli S, Mosca M, Maccauro G et al (2023) Adult acquired flatfoot deformity: a narrative review about imaging findings. Diagnostics 13(2):225 36673035 10.3390/diagnostics13020225 PMC9857373

[19] Oto M, Thabet A, Miller F, Holmes L (2011) Correlation between selective pedobarographic and radiographic measures in the assessment of surgically treated CTEV patients. Eklem Hastalik Cerrahisi 22(3):145–148 22085349

[20] Narang A, Sud A, Chouhan D (2021) Calcaneal lengthening osteotomy in spastic planovalgus feet. J Clin Orthop Trauma 13:30–39 33717872 10.1016/j.jcot.2020.08.024 PMC7920091

[21] Ursei M, Thevenin-Lemoine C, Lebarbier P (2016) Patología del pie en la parálisis cerebral infantil. EMC - Podología 18(3):1–12

[22] Macinnes P, Lewis TL, Griffin C, Martinuzzi M, Shepherd KL, Kokkinakis M (2022) Surgical management of pes planus in children with cerebral palsy: a systematic review. J Child Orthop 16(5):333–346 36238147 10.1177/18632521221112496 PMC9550996

[23] Rangasamy K, Ghosh AK, Ksheerasagar VP, Khatri JP, Gopinathan NR (2023) Effectiveness of lateral column lengthening in symptomatic flexible flatfoot of the pediatric and adolescent population: an updated systematic review. J Foot Ankle Surg 10(2):66–75

[24] Rajan L, Kim J, Fuller R, Cororaton A, Mizher R, Srikumar S et al (2022) Impact of asymptomatic flatfoot on clinical and radiographic outcomes of the modified lapidus procedure in patients with hallux valgus. Foot Ankle Orthop 7(2):24730114221099920 35615073 10.1177/24730114221099922 PMC9125072

[25] Li B, He W, Yu G, Zhou H, Xia J, Zhao Y et al (2021) Treatment for flexible flatfoot in children with subtalar arthroereisis and soft tissue procedures. Front Pediatr 9:656178 34095026 10.3389/fped.2021.656178 PMC8175848

[26] Aronson J, Nunley J, Frankovitch K (1983) Lateral talocalcaneal angle in assessment of subtalar valgus: follow-up of seventy grice-green arthrodeses. Foot Ankle 4(2):56–63 6642324 10.1177/107110078300400202

[27] Lin YC, Mhuircheartaigh JN, Lamb J, Kung JW, Yablon CM, Wu JS (2015) Imaging of adult flatfoot: correlation of radiographic measurements with MRI. Am J Roentgenol 204(2):354–9 25615758 10.2214/AJR.14.12645

[28] Oskoui M, Coutinho F, Dykeman J, Jetté N, Pringsheim T (2013) An update on the prevalence of cerebral palsy: a systematic review and meta-analysis. Dev Med Child Neurol 55(6):509–519 23346889 10.1111/dmcn.12080

[29] Rosenbaum P, Paneth N, Leviton A, Goldstein M, Bax M (2007) A report: the definition and classification of cerebral palsy April 2006. Dev Med Child Neurol 49(SUPPL. 2):8–14 17370477

[30] Turriago CA, Arbeláez MF, Becerra LC (2009) Talonavicular joint arthrodesis for the treatment of pes planus valgus in older children and adolescents with cerebral palsy. J Child Orthop 3(3):179–83 19308477 10.1007/s11832-009-0168-7 PMC2686809

