# Supplementary material for: Dynamic radiographic angle changes in planovalgus foot correction among children with cerebral palsy
Source: Arch Orthop Trauma Surg. 2026 Feb 19;146(1):65. doi: 10.1007/s00402-026-06206-6 (PMC12920777; doi:10.1007/s00402-026-06206-6)
Supplement: Supplementary file 1 — Supplementary Material 1 [file 402_2026_6206_MOESM1_ESM.docx]

**SUPPLEMENTARY MATERIAL**

**Supplementary Table 1****. Radiographic angles in the AP view commonly used to evaluate planovalgus foot in CP.**

| **Angle** | **Radiographic AP View** | **Normal Value** | **Key Diagnostic/ Severity Findings** | **Clinical Correlations & Management** |
| --- | --- | --- | --- | --- |
| **AP talocalcaneal (Kite´s) angle.** It is formed by the intersection of the talus and calcaneus axes (4). | 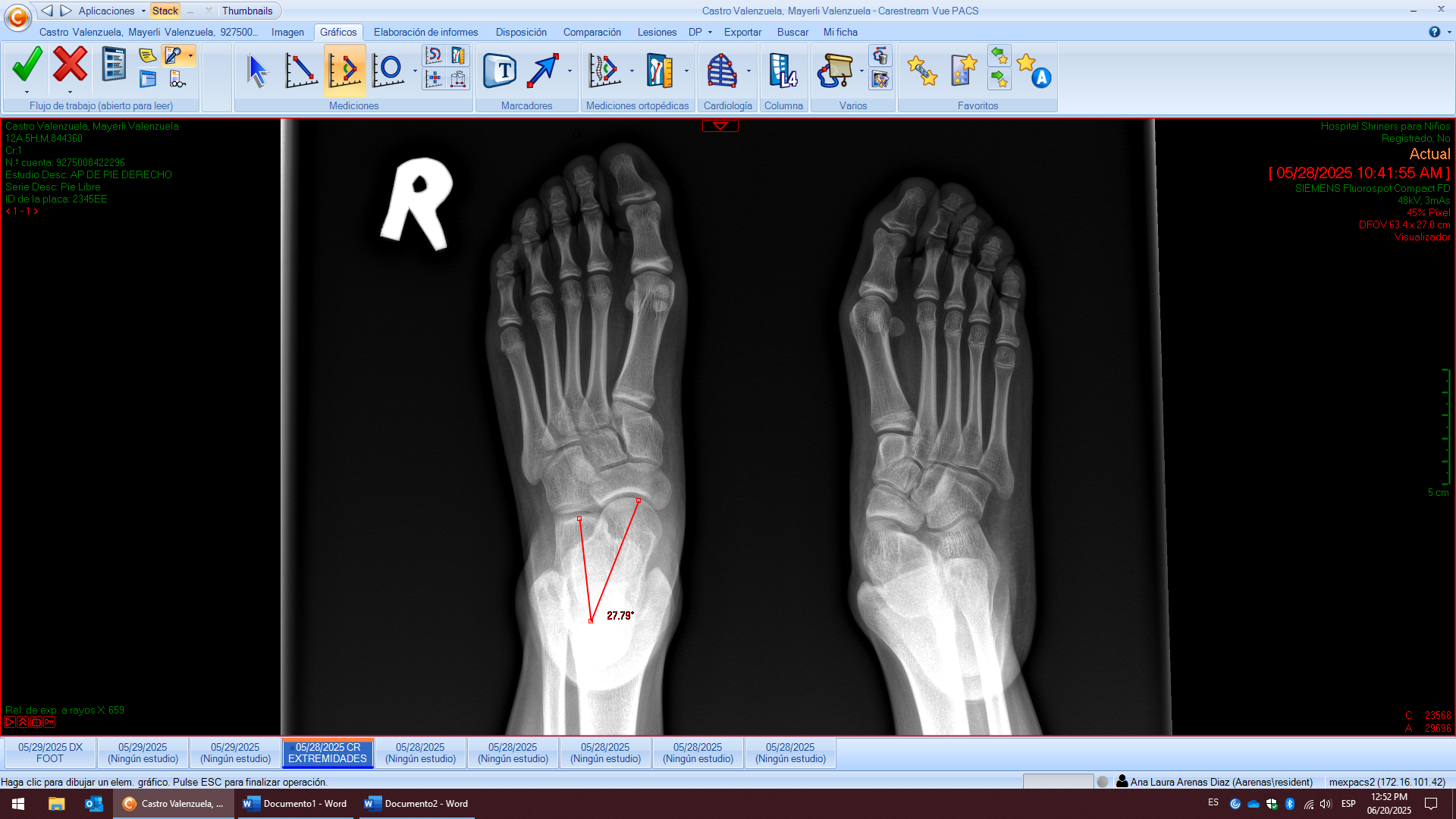 | 20 to 40°  (4)  Quantifies hindfoot valgus and forefoot abduction; excessive values suggest planovalgus deformity. | In CP planovalgus deformity, the talus is medially subluxed and the calcaneus is everted, resulting in a wider Kite's angle. A clinical review indicates that a Kite's angle over 25 ° suggests a pronated foot (9). | Studies show pre-operative angles in severe CP cases can be around 37 to 38 °, improving to about 22 ° after surgical correction. In a long-term study of talonavicular arthrodesis for CP planovalgus, the mean angle improved from approximately 37.6 ° pre-operation to 22.6 ° post-operation (p<0.01), approaching the normal range (10). |
| **AP talar–first metatarsal angle (AP talo–1st MT).** The angle between the talus and the first metatarsal. This angle indicates the forefoot's abduction relative to the hindfoot (11). | 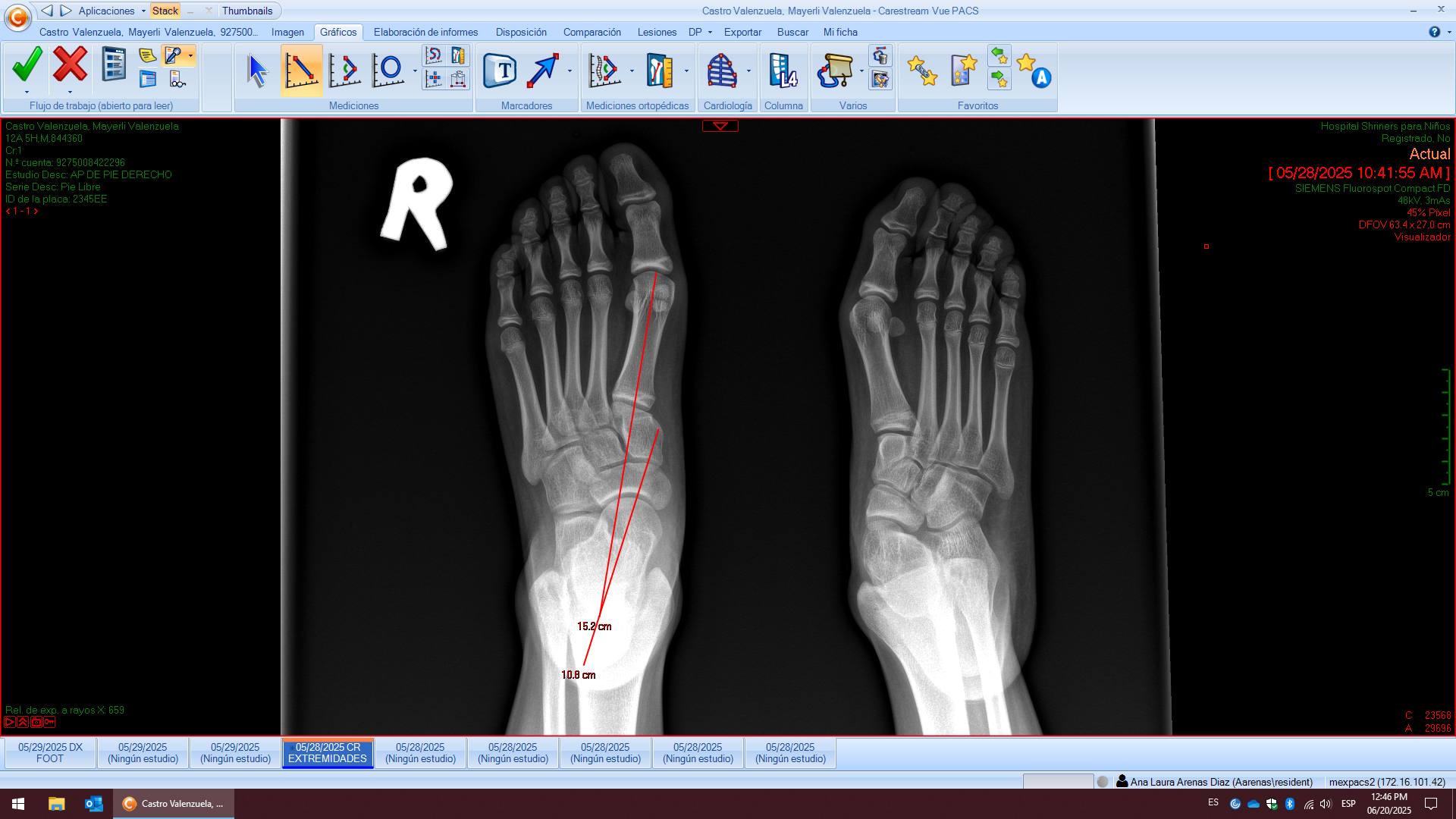 | 0° and 5°  (11) | In planovalgus, the forefoot abducts and the talus points medially, resulting in a positive angle. Children with CP often show a significantly increased angle, indicating lateral drift of the forefoot (11). | A study showed that the median preoperative angle in symptomatic planovalgus feet was about 30°, improving to normal post-surgery (12). A threshold of 23° was identified as significant; if the angle exceeds 23° preoperatively, a standalone calcaneal osteotomy may be insufficient for correction. In these cases, additional procedures like posterior tibial tendon reefing or talonavicular joint fusion are often necessary (13,14). Overall, the AP talo–1st MT angle aids in diagnosing flatfoot and determining its severity, with angles over 20° to 25° indicating more severe deformities that may require combined surgical approaches (15). |
| **Talonavicular Coverage/Uncoverage Angle,** measures how well the navicular bone covers the talar head in an AP radiograph (16). | 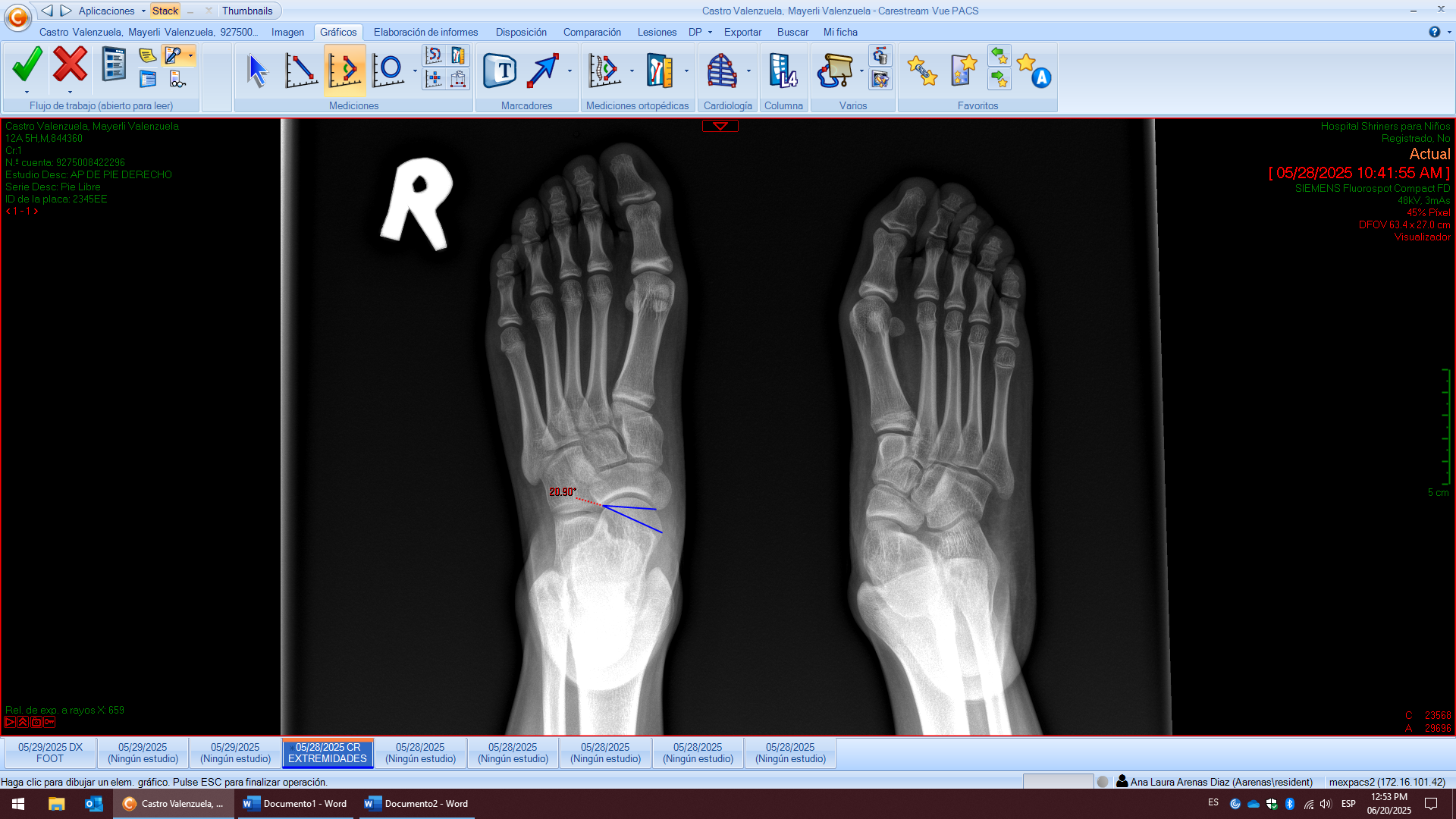 | <20% uncovered (16) | Look et al. (2021) identified the TN coverage angle as a key measure for classifying the severity of pes planovalgus. In their analysis of 395 CP feet, this angle effectively distinguished between mild, moderate, and severe cases (17). A long-term surgical study reported that the mean TN coverage angle improved dramatically from about 37 degrees before surgery to nearly 2 degrees after talonavicular fusion in CP patients (18). | Measuring TN coverage on radiographs requires a clear view of the talar head and navicular interface. While this is usually feasible in older children, very young patients with CP may have incomplete navicular ossification. Consequently, Min et al. (2020) excluded TN coverage angles and naviculocuboid overlap from their analysis for children under about 4 years. For fully ossified feet, the TN coverage measure is typically reproducible and has been widely adopted in evaluations (3–16).  If there is significant uncoverage of the talonavicular joint (over 40%), a talonavicular arthrodesis would be recommended or a medial column procedure. Kim et al.(5) noted that a naviculocuboid uncoverage greater than 72% is a critical threshold, beyond which a calcaneal osteotomy alone may not provide adequate correction (11,19). |
| **Calcaneus–5th Metatarsal Line Angle. T**he angle between a line drawn along the lateral border of the calcaneus and a line along the lateral border of the fifth metatarsal (20). | 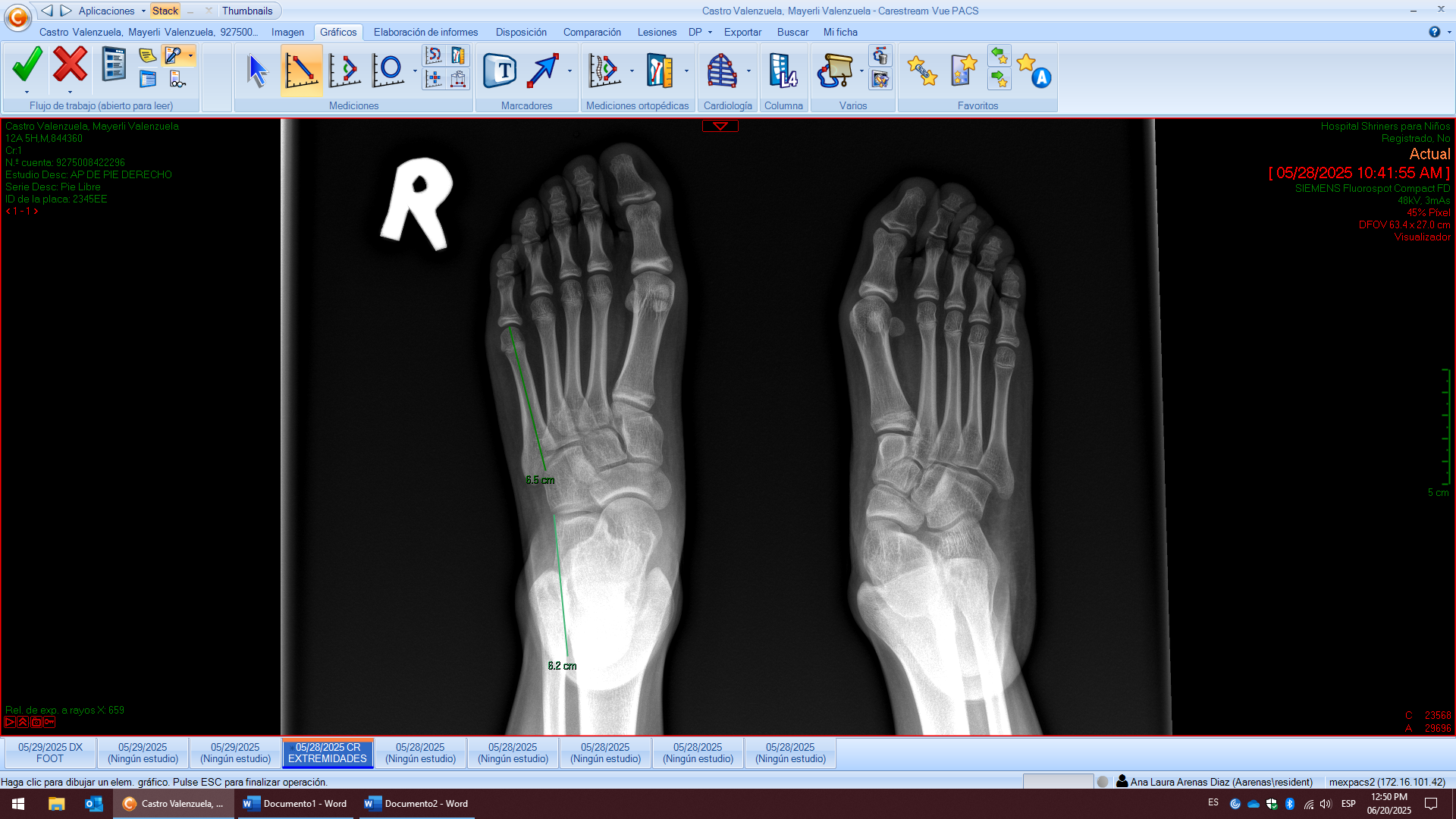 | Close to 0°  (4) | In individuals with planovalgus deformities, elevated C5M angles are associated with more severe transverse plane deformities. A significantly positive angle can destabilize the midfoot joints, making push-off difficult and reducing gait efficiency. Furthermore, forefoot abduction can lead to increased energy expenditure during walking and diminish lever-arm efficiency in these cases (19–22). | A focused review of lateral column lengthening outcomes revealed that only a few studies reported the AP angle. Pooled data showed that the C5M angle averaged about 21.4° (11° to 30° range) preoperatively and reduced to 3° to 4° after surgery. This significant decrease indicates that lateral column lengthening effectively realigns the forefoot and hindfoot, restoring the lateral border of the foot to a straighter position (23,24). |

**Table 1**. This literature review focuses on radiographic angular parameters in the anteroposterior view that are applied in the assessment of structural alterations associated with planovalgus foot in CP, aiming to guide clinical decision-making.

**Supplementary Table 2.** **Radiographic angles in the lateral view are commonly used to evaluate planovalgus foot in CP.**

| **Angle** | **Radiographic Lateral View** | **Normal Value** | **Key Diagnostic/ Severity Findings** | **Clinical Correlations & Management** |
| --- | --- | --- | --- | --- |
| **Moreau–Costa–Bartani (Medial Longitudinal Arch) Angle.** This angle is formed by two lines along the medial side of the foot: one line connects the lowest point of the calcaneus to the lowest point of the talonavicular joint, and the second line extends from that talonavicular point to the inferior aspect of the first metatarsal (25). | 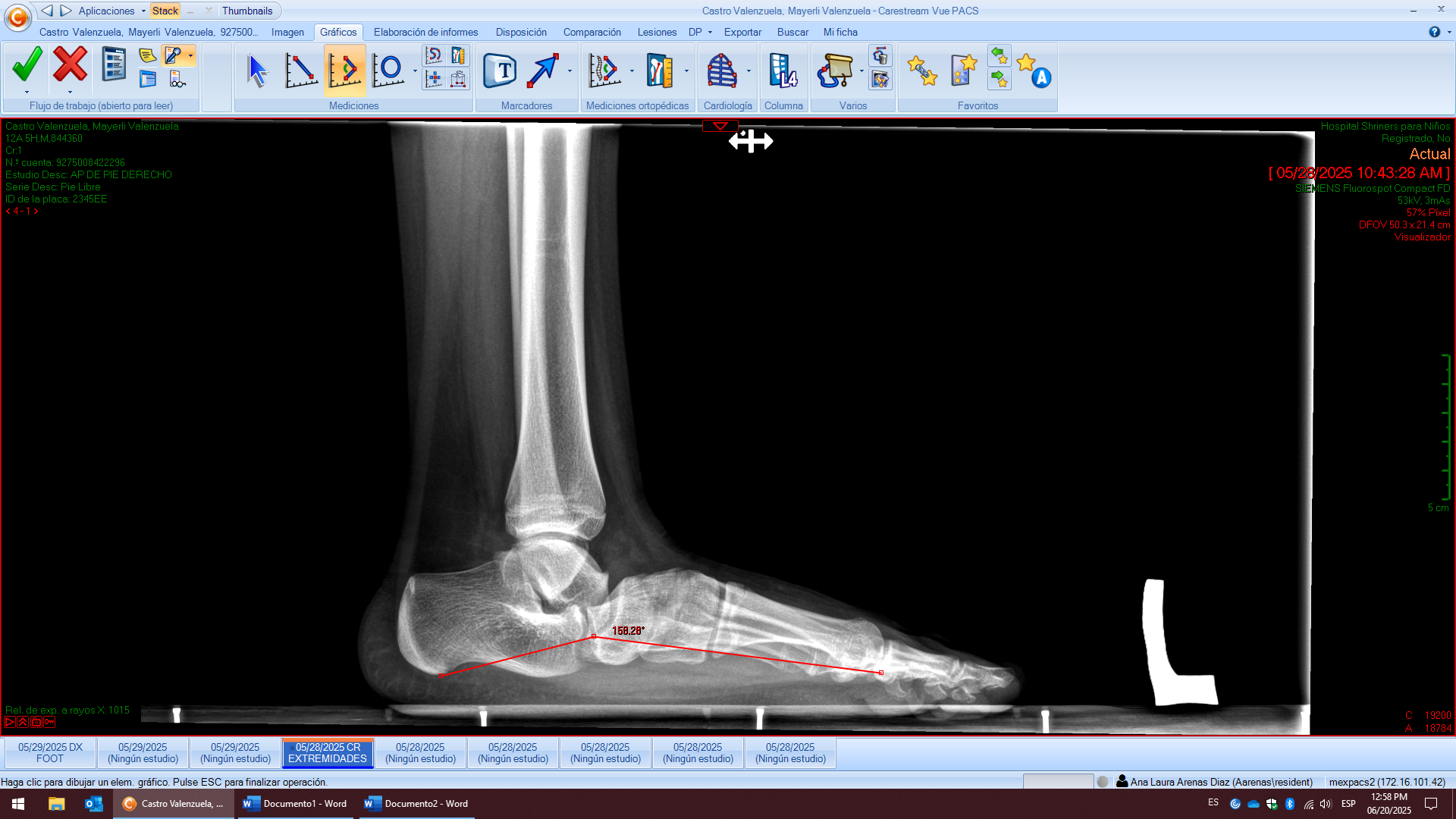 | Around 120°  (16) | In CP, the Costa-Bartani angle is typically significantly increased, indicating the presence of the planovalgus deformity (greater than 130°) (16–26). | A 2022 study examining talonavicular fusions found that the preoperative Moreau–Costa–Bartani angle averaged 167° in the planovalgus feet of CP patients. After a 10-year follow-up after surgery, this angle improved to approximately 146° (p<0.01) (10). This significant change, from 167° to 146°, reflects a restoration of the medial arch; however, an angle of 146° still remains slightly above the normal range, suggesting a persistent tendency toward flatfoot (5,10,11,13–16). |
| **Meary’s Angle (Lateral Talar–First Metatarsal Angle).** The angle formed between the long axis of the talus and the long axis of the first metatarsal in a weight-bearing lateral view (23). | 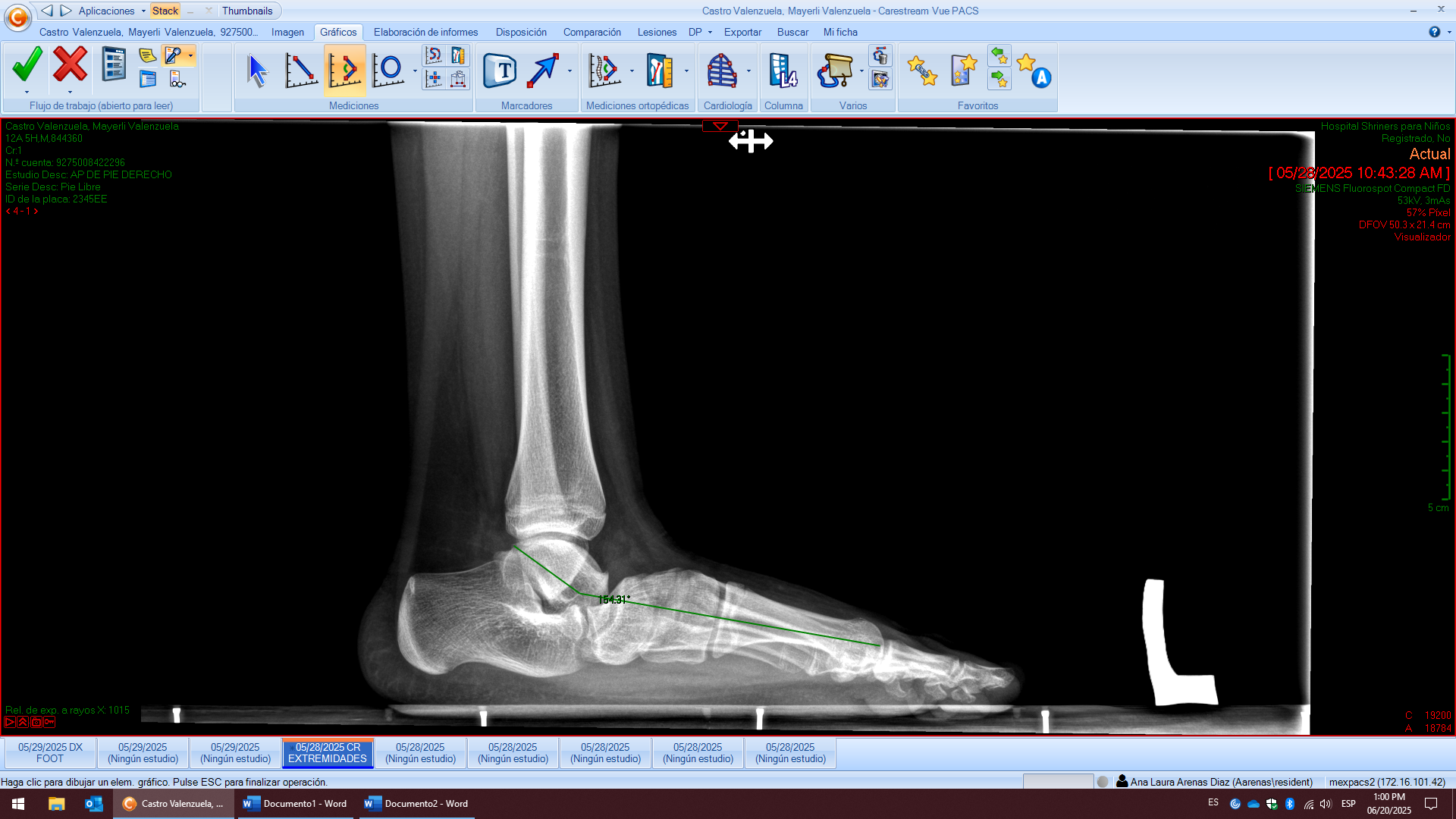 | Approximately 0°  (23) | Clinically, a Meary’s angle greater than 4°–5° is typically considered abnormal in children, while an angle exceeding 10° is regarded as clearly pathological (16).  In CP planovalgus, Meary’s angle can be extremely high due to severe midfoot collapse (10). | The 2022 talonavicular arthrodesis study found that patients with level GMFCS III- IV had an average Meary’s angle of 37.9° before surgery, a significant deformity. Postoperatively, this angle improved to around 8.4°, indicating that the surgery effectively corrected the arch alignment from extreme flatfoot to mild flatfoot (10).  Similarly, Kim's 2013 study on calcaneal lengthening noted a critical lateral talo-first metatarsal angle of 36°. If this angle exceeded 36° preoperatively, isolated calcaneal osteotomy often under-corrected the arch, suggesting the need for additional procedures. This aligns with the idea that angles greater than 30°–35° indicate a severe deformity that requires more than just hindfoot repositioning (14,16,22,23). |
| **Lateral Talocalcaneal Angle. It** is measured between the axes of the talus and calcaneus on a lateral radiograph. This angle reflects the subtalar alignment in the sagittal plane (24,25,27). | 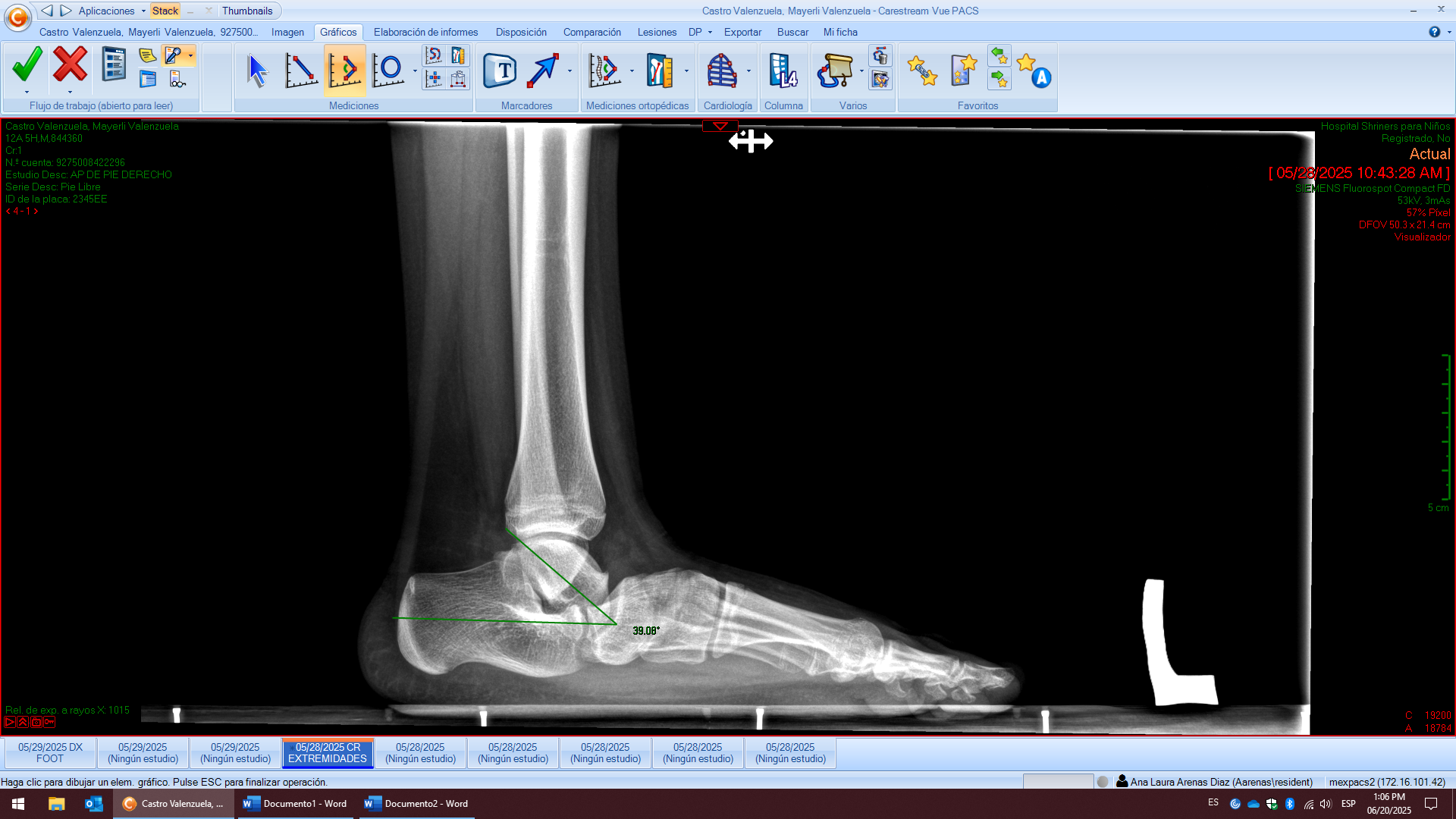 | 30° to 40°  (27) | Typically, the angle is elevated (40–50°) but can normalize (25–35°) after correction in CP  (26,27). | Studies have reported an abnormal increase in the lateral talocalcaneal angle in individuals with CP. For instance, a preoperative average angle of 42.2° was noted in patients with severe planovalgus feet, which decreased to 25.3° after undergoing talonavicular arthrodesis (5). Similarly, in another study (Aronson et al., 1983), lateral talocalcaneal angles of approximately 45–50° were observed in valgus feet, which were reduced to around 30° after subtalar stabilization (27). |
| **Calcaneal Inclination (Pitch) Angle. T**he angle formed between the inferior calcaneus and the horizontal plane, which is the foot–floor interface, as seen on a lateral weight-bearing radiograph. This angle reflects the tilt of the calcaneus and serves as an indicator of arch height (16,28). | 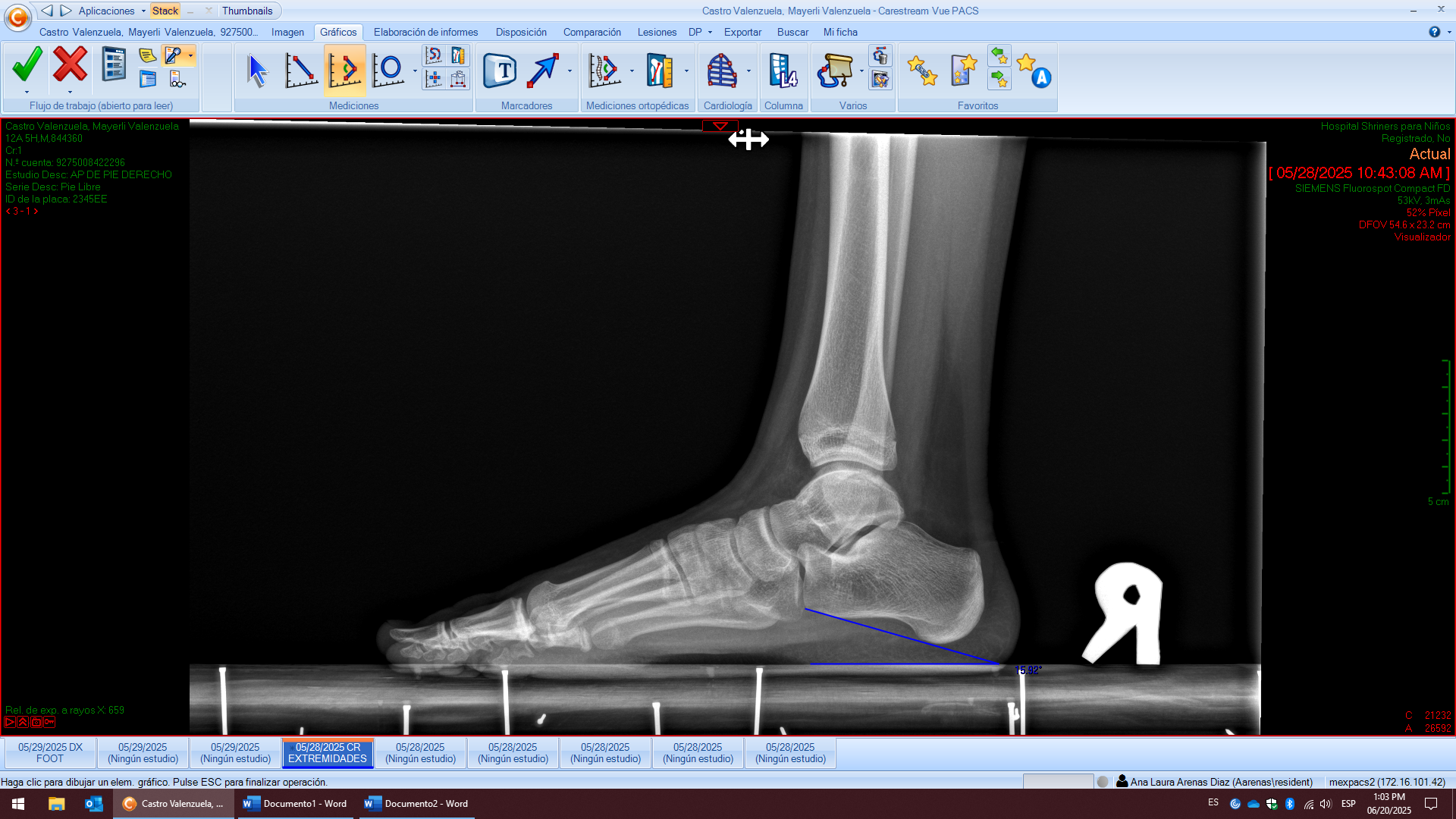 | 18 to 30 °  (28) | In CP planovalgus, the calcaneal pitch is often significantly reduced, sometimes approaching 0° or negative in severe cases.  Colleagues sometimes use thresholds like <5° to define severe flatfoot (28). | A study on CP planovalgus feet that underwent TN fusion showed an average calcaneal pitch of 3.2 ° before surgery, improving to 13.2° at a 10-year follow-up .^6,18^ Although 13°is still slightly low, this change was statistically significant (p < 0.01) (29). Another study on calcaneal lengthening also noted an average increase of about 9°in calcaneal pitch post-surgery, but preoperative values were not provided (5,27,28). |

**Table 2.** This literature review focuses on radiographic angular parameters in the lateral view that are applied in the assessment of structural alterations associated with planovalgus foot in CP, aiming to guide clinical decision-making.

| **Surgical variable** | **n (%) of feet (n = 132)** | **Description** |
| --- | --- | --- |
| Talonavicular management | 82 (62.1%) reefing; 50 (37.9%) arthrodesis | Based on joint stability and deformity rigidity |
| Bone graft use | 33 (25.0%) | Autologous tricortical iliac crest graft |
| Fixation method | 116 (87.9%) K-wire; 16 (12.1%) screw or plate | Chosen according to osteotomy stability |
| Joint–osteotomy distance | Mean 11.0 mm (range 7.05–15) | Measured intraoperatively |
| Osteotomy length | Mean 7.6 mm (range 5.5–28) | |
| Immobilization type | 77 (58.3%) cast; 55 (41.7%) splint | Duration: 6 weeks |
| Reintervention performed | 20 (15.2%) | - |

**Supplementary Table 3**. Surgical variables and intraoperative characteristics of the 132 feet included in the study.

| **Reinterventions performed** | **n=20** |
| --- | --- |
| Talonavicular Arthrodesis | 10 (50%) |
| Revisions of calcaneal lengthening and talonavicular arthrodesis | 6 (30%) |
| Talonavicular and calcaneocuboid arthrodesis | 3 (15%) |
| Revision of calcaneocuboid lengthening | 1 (5%) |

**Supplementary Table 4**. Types of Reinterventions performed

**Supplementary material cases presentation**

To provide further illustration and context, two radiographic cases from the studied cohort are presented below (Cases 1 and 2).

**Case 1.** An 11.9-year-old male with spastic diplegia (GMFCS level III) presented with a non-rigid spastic planovalgus foot. Surgical treatment consisted of calcaneal lengthening combined with talonavicular joint reefing.


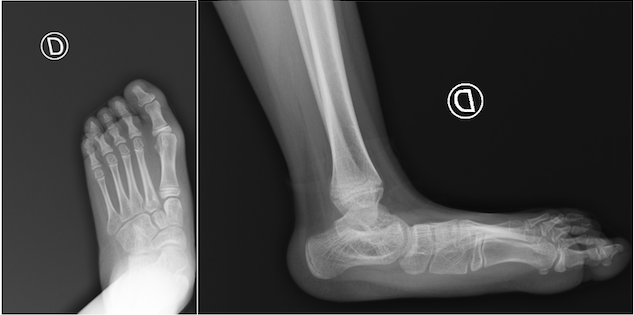
 (a)


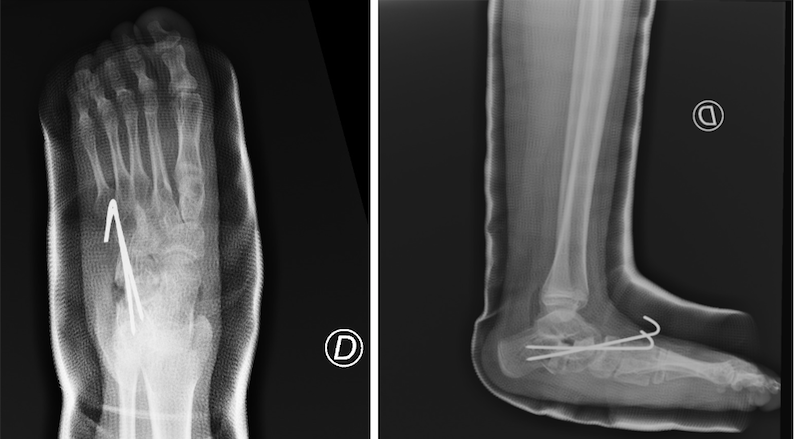
 (b)


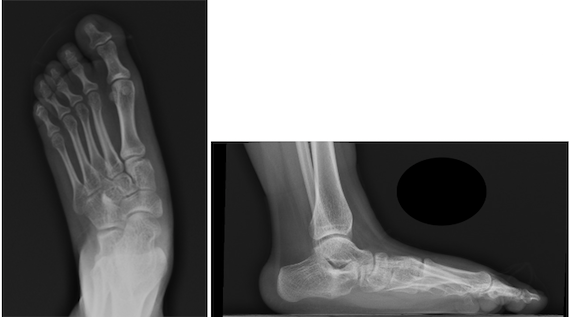
 (c)

**Case 1. Figure a.** Preoperative AP and lateral weight-bearing radiographs of the right foot demonstrate the deformity, with loss of normal foot alignment and angular parameters. **Figure b.** Immediate postoperative AP and lateral views show restoration of foot alignment and correction of the abnormal angles. **Figure c.** AP and lateral radiographs at 2-year follow-up reveal recurrence of the planovalgus deformity, with partial loss of the previously corrected radiographic angles, indicating a gradual deterioration of surgical correction over time.

**Case 2.** A 14.6-year-old female with spastic diplegia (GMFCS level III) presented with a flexible spastic planovalgus deformity of the left foot. Surgical management included calcaneal lengthening combined with talonavicular arthrodesis


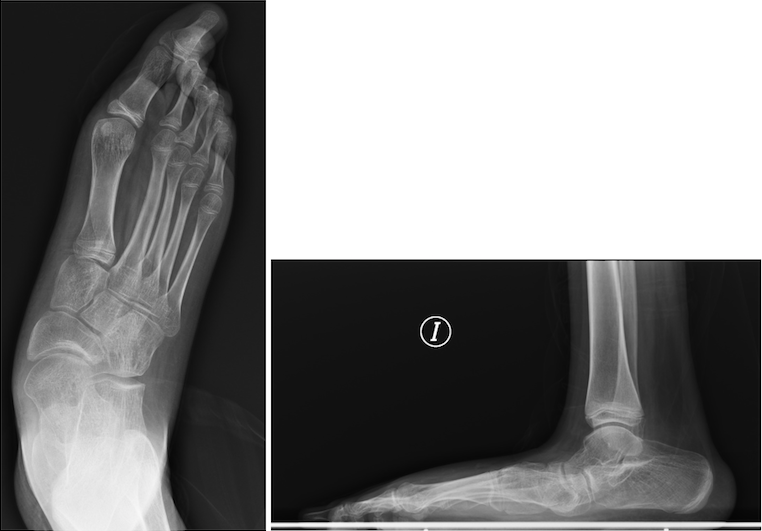
 (a)


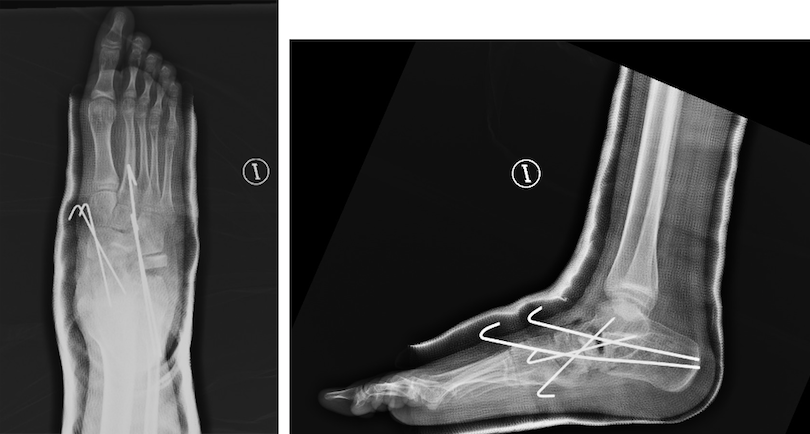
 (b)


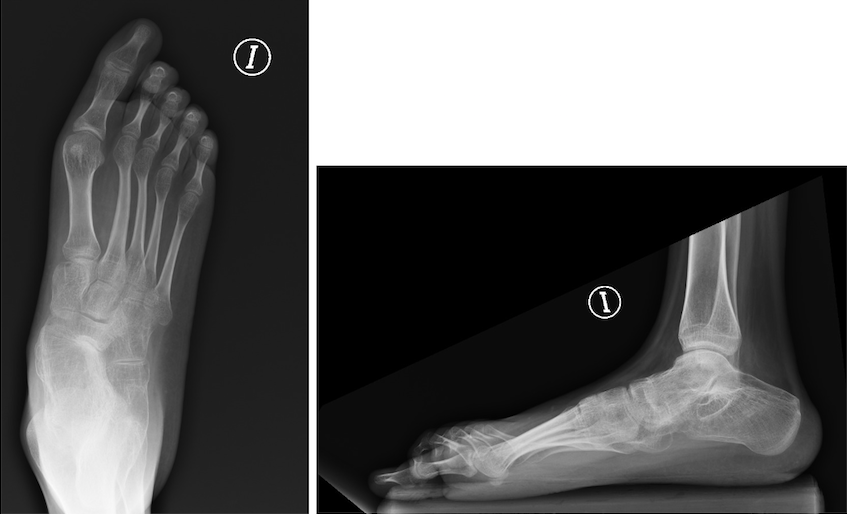
 (c)

**Case 2. Figure a.** Preoperative AP and lateral radiographs of the left foot show marked deformity, with loss of normal alignment, talonavicular uncoverage greater than 25%, and articular surface changes at the talonavicular joint. **Figure b.** Immediate postoperative images in the same views demonstrate restored alignment. Talonavicular fusion was stabilized with 1.6 mm Kirschner wires due to observed low bone quality. **Figure c.** Radiographs at two-year follow-up reveal partial loss of correction, along with degenerative changes in the talocalcaneal and calcaneocuboid joints.
